# The case for openness in engineering research

**DOI:** 10.12688/f1000research.14593.2

**Published:** 2018-10-11

**Authors:** Devin R. Berg, Kyle E. Niemeyer

**Affiliations:** 1 Engineering & Technology Department, University of Wisconsin-Stout, Menomonie, WI, 54751, USA; 2 School of Mechanical, Industrial, and Manufacturing Engineering, Oregon State University, Corvallis, OR, 97331, USA

**Keywords:** open science, open engineering, engineering, open access, research dissemination

## Abstract

In this article, we describe our views on the benefits, and possible downsides, of openness in engineering research. We attempt to examine the issue from multiple perspectives, including reasons and motivations for introducing open practices into an engineering researcher's workflow and the challenges faced by scholars looking to do so. Further, we present our thoughts and reflections on the role that open engineering research can play in defining the purpose and activities of the university. We have made some specific recommendations on how the public university can recommit to and push the boundaries of its role as the creator and promoter of public knowledge. In doing so, the university will further demonstrate its vital role in the continued economic, social, and technological development of society. We have also included some thoughts on how this applies specifically to the field of engineering and how a culture of openness and sharing within the engineering community can help drive societal development.

## Introduction

Working openly should be the default mode of science—after all, how can we advance knowledge “by standing on the shoulders of giants” ^a^ if we cannot access or see those shoulders? As there is no clear consensus as to how to define open science ^1^, this paper operates on the following definition, which was first laid out by Niemeyer ^2^ and represents a synthesis of other available definitions ^b^:

Open science, or more broadly open research, describes the activity of performing scientific research in a manner that makes the products and findings accessible to anyone. This includes sharing data openly (open data), publicly releasing the source code for research software under a permissive license (free and open-source software), publicly releasing the designs of research hardware under a permissive license (free and open-source hardware), and making the written products of research openly accessible (open access).

The field of engineering provides an interesting case study for examining the impacts of open practices since engineering touches every aspect of human life. Engineering research is inherent to the development of goods and products such as medical devices and pharmaceuticals, so issues around the protection of intellectual property and innovation draw stark contrasts, for some, with the tenets of open science. On the other hand, work being done in the free and open-source software (FOSS) and free and open-source hardware (FOSH) movements can enable us to engineer the tools of modern scientific discovery, greatly reducing the costs of scientific research ^3^. These two movements derive from two complementary principles, that the software source code and hardware design should be openly released and licensed for reuse and modification ^4, 5^.

In a time of constrained university budgets, which are not expected to improve as long as most public universities rely heavily on public funding from state and federal sources, many universities are being forced to evaluate their institutional priorities ^6^. For some, particularly state universities subject to the whims of state legislation, this could mean abandoning the pursuit of fundamental or basic knowledge generation in favor of marketable vocational training models that cater more directly to industry needs. While this model is in line with the Morrill Act of 1862, which created land-grant colleges and underpins the missions of many US institutions, the university has evolved since that time to encompass a much greater proportion of the economic development of the country ^6^. Despite the challenges faced by institutions today, our opinion is that it is critical for the university to continue to position itself as a center of societal development—economically, technologically, and socially. Additionally, under our interpretation of the mission of the land-grant university program, the university should push this model further towards positioning itself as the main driver of social and technological innovation in its geographic region. To achieve this, it is necessary to organize and market the business of the university, as clearly as possible, as a service provider to many relevant stakeholders. This can be best accomplished by disseminating and distributing the products of university activities as widely as possible through open-access publishing, open research, and open innovation, and further demonstrating the impact that these products have on local, state, national, and international populations. As stated by Ashley Farley of the Gates Foundation, “Open research should be the norm. Knowledge should be a public good” ^7^.

This article seeks to motivate the importance of open science, particularly for engineering research, and synthesizes our earlier white papers ^2, 8^ separately discussing this topic. We discuss the importance of open science, describe how practicing open science increases the societal impact of research, provide recommendations for researchers to practice open science, summarize challenges to working openly, and conclude with some recommendations for university leaders to promote open (engineering) research. While many of the issues discussed herein apply more broadly beyond the field of engineering, we have elected to focus our discussion on engineering rather than speak for other disciplines.

## Importance of open science

Transforming research communities from traditional, closed environments to open ones is important for a number of reasons, including (but not necessarily limited to) the following seven. McKiernan *et al.* ^9^ further discuss these and additional benefits for researchers working openly. Tennant *et al.* ^10^ review in detail the benefits of open-access publications to academics and society.

**Value and credit:** Conducting open research entails recognizing the value of all of the products of research. This includes all the final, polished products of that work—papers, software, and data—as well as the failures and null results ^11, 12^. The dissemination of these artifacts may on occasion comprise an act of humility, but ultimately it recognizes that each of these items is a piece of the research process and that even your failures have value in the lessons you learned and can be passed on to others. Often researchers will contribute significant time and effort to developing products other than journal articles. If this work is not properly credited, career progress can be stifled as a result. One example of this is the development of scientific software, which can support the work of other researchers around the world ^13– 16^. If this activity is not supported in terms of career progression, the entire research community suffers as a result.

**Accessibility:** Openness in research ensures that research products, particularly written output, remain accessible to all. This includes the research community, funders, policy makers, and the general public. Accessibility of research products is particularly important for publicly funded research—since the public paid for the research, the public should have access to it and be able to benefit from it. This does not prevent innovators or other parties from developing commercial intellectual property based on the findings, but ensures that the original discovery, when funded by the public, remains accessible to all. While nearly 50% of all published work is available freely through open-access sources, institutional archives, or online social networks, this percentage is notably lower in engineering: approximately 35% ^17^. However, Piwowar *et al.* ^18^ found that this percentage drops below 20% when not considering articles self-archived on author websites, which can lack assurance of long-term availability.

**Reproducibility:** Releasing products of research, including software and data, helps enable reproducibility. This is particularly true for computational science, where a written description of methods can never describe an approach as completely as the source code ^19^. In general, access to research software used to perform a computational study, or the data from an experimental study, should enable others to reproduce the findings of the original researchers. However, open science is a necessary but not sufficient aspect of reproducibility, as it can be challenging to reproduce or replicate results even with available research software and data ^20, 21^.

**Recognition:** As a selfish motivation, performing research openly helps increase the recognition received by the work. Studies have shown that open-access papers are cited more in most research fields. In engineering, open-access papers are cited around 1.5 times more often than non-open-access papers ^17, 22^. Similarly, papers with associated open data were cited 9–50% more than those without ^9, 23^. Vandewalle ^24^ showed that papers in the image-processing field receive up to three times the number of citations when source code is made available. We must note, however, that the concept of recognition should not be solely regarded through the measure of citations. The true societal impact of the work is likely more important but also more difficult to quantify ^25^. This point is discussed further in the following section.

**Establish priority:** Some researchers hesitate to embrace open science out of a fear of being “scooped,” where competitors will use some findings, software tools, or data made available and then publish first. However, contrary to this belief, practicing open science can actually prevent being scooped: releasing preprints can establish priority of discoveries or techniques prior to the publication of a traditional peer-reviewed journal article ^26, 27^.

The peer-review and editorial process of such papers can take many months or years, but journal articles are still necessary for research findings to be considered valid (and for researchers to receive credit). Publishing a preprint of an article publicly time-stamps the work, even as it undergoes peer review and possible revision.

**Encourages trust:** Embracing openness in scientific research can help encourage other researchers to trust published results, by giving the ability to inspect data or software. Soergel ^28^ estimated that 5–100% of computational results given by software may be incorrect or inaccurate. While simply releasing source code openly will not solve this problem, this is a necessary step towards verification and reproducibility.

**It’s nice:** In addition to the above benefits, sharing products of research openly is kind to colleagues and the greater research community, as it prevents people from wasting time by unnecessarily repeating work. For example, many graduate students begin working on their dissertation research by attempting to reimplement another group’s methods and reproduce some of their published results. However, lacking access to software source code or datasets can hinder this work. As a result, significant time can be wasted guessing about minor implementation details or inputs not discussed in the corresponding published papers. This can be avoided by sharing the source code and data, which would allow these junior researchers to more quickly move on to new work. Graduate students and other researchers constantly face similar challenges that could be avoided by greater openness in research.

## Openness increases societal impact of research

Many published journal articles go unread, even in their topical domains. One study of citation rates found that 27% of papers published in the natural sciences and engineering go uncited ^29c^. Those who do read most papers likely come from research institutions similar to those of the authors, even if the findings could be impactful beyond these confines, for example by leading to policy changes or technological solutions for humanitarian purposes. In part, this is due to the challenging technical content, jargon, and niche topics—but it is also due to a lack of access to the journals where most research findings reside. Making the content of these papers actually understandable or digestible by most potential readers is another challenge.

Considering the high and ever-increasing cost of scholarly journal subscriptions ^30^, research results should not be limited to those with the means to purchase access. By self-archiving (i.e., green open access) or publishing articles in open-access journals, researchers can ensure access for all members of society, including policymakers, funders, members of the media, entrepreneurs, and the general public—as well as scientists and engineers in the Global South.

Furthermore, being more open with all outputs of research (e.g., papers, software, data) could help improve the general public’s perception and trust in scientific research. Simply making research products available will not solve all of these problems—for one, it will not sway those who strongly believe ideas contrary to fact. However, ensuring everyone has access to the data researchers generate and analyze, and the software tools on which we rely, could eliminate one major barrier to trust in our findings ^31^.

Looking specifically at the field of engineering, we can also find examples of the positive effects of open knowledge dissemination. According to Chris Ategeka, founder of Health Access Corps, “Patenting a social-impact product hinders scale, ultimately obstructing the maximum impact that particular product would have in the world if it was open source” ^32^. Thus, the clear benefit of using open research and development practices is achieving greater impact with your research products. Indeed, from an ethical standpoint, engineers working on technologies targeted at the world’s most vulnerable communities should prioritize the open release of these technologies. The counter argument to this is that, through patenting, the entrepreneur can more easily market and sell their product in developed markets, which could then increase their ability to affect change by subsidizing their efforts in developing nations. This situation may hold true for products with broad appeal and therefore it is necessary for the inventor to assess which path will produce the greatest impact. (This assumes, also, that we encourage and reward *impact*.) It could be argued that in the majority of scenarios, open dissemination will yield greater impact through simplified adoption and adaptation by others, especially if the front-end development activities are incentivized in other ways. Indeed, we can begin to measure this impact through metrics such as download counts for open source software and hardware projects to demonstrate market penetration. From another perspective, there is some evidence that the pursuit of patents for university research slows innovation ^33^, while the return on investment for publicly and privately funded research is high ^34, 35^. Similarly, the use of FOSS and FOSH may actually increase the return on investment when used to their full potential ^36^.

## Open science and the scholar’s research agenda

For the new researcher looking to build their profile and develop their research agenda, we present a vision and plan for performing research openly, synthesized from literature practices and advice. The ideas presented here are heavily inspired by examples from others active in this area such as Lorena Barba’s Reproducibility PI Manifesto ^37^, the Peer Reviewers’ Openness Initiative ^38^, and others. While these exemplars provide useful case studies, it is important to emphasize that each individual must define for themselves a workflow that works for them. Sometimes it is enough to simply be more open than the current norms in their field.

Many fields within engineering lack reputable open-access journals; indeed, only 17% of published manuscripts in engineering can be accessed by the public for free legally, while some sub-disciplines, such as chemical engineering, are lower at 9% available ^18^. Thus, the engineering researcher looking to publish in open-access venues can quickly become discouraged. Early career researchers looking to make their work available while operating within this research environment can take simple steps such as submitting preprints of any publications to the engrXiv ^d^, and deposit (otherwise non-accessible) conference papers or slide decks on Figshare ^e^ or Zenodo ^f^.

For the researcher looking to develop their open workflow further, we recommend the following steps:

- Make all written research products openly accessible, either through green or gold open-access avenues. For fields that lack recognized, fully open-access journals, this objective can be met by submitting preprints to services such as arXiv, engrXiv, PeerJ Preprints, Figshare, or Zenodo, depending on the topic. Conference papers, when not submitted to an open venue, can also be made openly available. Where possible, release all preprints under the Creative Commons Attribution (CC BY) license. ^g^ If funds are available through research grants or library sources designated to support open-access publishing, a researcher may choose to follow the hybrid gold open-access model by paying a non-fully open journal to make a paper accessible. Note, however, that the fees associated with hybrid open-access journals are detrimental to researchers at smaller institutions ^39^ and thus this model should not be viewed as the solution in keeping with the goals of the open-access movement.
- Any new research software developed should be done openly (e.g., on GitHub), released publicly under a permissive license, such as the BSD 3-clause license, and cited appropriately in any publications that rely on it ^16^. The Git version-control system (or equivalent) should be used to track the history of software projects, and software releases associated with publications or data should be archived (with DOIs) using Zenodo. In addition, implementation details should be described as thoroughly as necessary to reproduce the work. Similarly, the design of any research hardware that is developed should be publicly released under a permissive license.
- When making use of existing research software and/or hardware, use FOSS/FOSH whenever possible to permit the greatest reproducibility.
- All data generated through research, when serving as the basis for a publication, should be archived publicly and cited appropriately in manuscripts or other documents ^h^. This data may also include figures and the plotting scripts that produce them, which can then be shared under a CC BY license and cited where appropriate.

As a means of supporting these efforts, researchers should take care to implement these policy statements by incorporating them into funding proposals, for example in Data Management Plans. Note that policies where data and code are made “available upon request” are generally not sufficient for reproducibility ^40^.

Several community efforts have developed in recent years with the goal of defining and supporting open science practices. Some examples include the Workshop on Sustainable Software for Science: Practice and Experiences (WSSSPE) series ^41^ and the FORCE11 Software Citation Working Group, which developed the Software Citation Principles ^16^ with the goal of standardizing software citation to help ensure authors/developers receive academic credit for their work in releasing open research software. On the publishing side, community-driven research journals have been built to promote open publishing practices. Some examples include the *Journal of Open Source Software* ^i42^ and the *Journal of Open Engineering* ^j 43^. Similarly, engrXiv, an open archive for engineering publications, has been developed to serve the engineering community, inspired by the success of arXiv.

## Challenges to performing open science

The primary challenges facing those individuals interested in conducting open research generally involve incentives (or the lack thereof) and restrictive policies maintained by traditional publishers, in addition to the lack of a culture of sharing within the researcher’s disciplinary field. First, researchers are often pressured to carefully consider the venue in which they publish their work and to select only those that are “well established” and “high impact.” However, if these venues are not amenable to open research activities such as the posting of preprints, these challenges disincentivize those activities. To remedy this, the research community must continue to pressure publishers to modify their copyright transfer policies. Some progress has already been made in this effort through policies from funding sources such as the National Institutes of Health ^k^, the National Science Foundation ^l^, the Bill & Melinda Gates Foundation ^m^, and the Wellcome Trust ^n^ as well as from research institutions who require deposition in a repository. More information on these policies can be found on the Registry of Open Access Repository Mandates and Policies ^o^. Additionally, authors themselves can in some cases work with publishers to modify the standard publisher copyright transfer agreements allowing the author to retain more rights ^p^.

Additionally, promotion and tenure requirements typically focus exclusively on the final published manuscript and associated metrics, neglecting other research outputs such as code, data, solid models, etc., and their associated impacts. Some institutions actively discourage making these alternative research products available due to idealistic dreams of future income generation from licensing revenues. However, in reality, the majority of universities *lose* money through their technology transfer offices, since translation of university intellectual property to commercial success is generally poorly realized ^44, 45^. Instead, institutions may pursue alternatives which promote universal knowledge dissemination as a mechanism to create impact from university research outcomes as opposed to monetary aims. Ultimately, it is likely that societal pressure is necessary to push more institutions to participate in such initiatives. For that to happen, the public first needs to be aware of the possible benefits of broad knowledge dissemination and needs to experience those benefits first hand. Researchers may even see benefits in terms of their scholarly productivity, as Frankenhuis and Nettle ^46^ argue that open-science practices may actually increase creativity and researcher output.

The challenges impeding greater adoption of open-science practices are mainly institutional and cultural, rather than technical. General venues for sharing and developing the products of research openly abound these days, with the availability of services like arXiv, engrXiv, and PeerJ Preprints for ensuring open access of publications; repositories like GitHub for developing (and version-controlling) research software openly; and data and software archives like Zenodo and Figshare, which practically have no file size limitations ^q^. Of course, some technical problems remain: How do we make results of computational science, particularly when it involves demanding high-performance computing resources, truly reproducible? How can we cite software and data consistently, when the version might change regularly? How can open practices be integrated into a researcher’s workflow without further straining the researcher’s already overburdened time?

As cultural inertia and lack of institutional recognition/rewards pose significant challenges to increased openness in science, the biggest barrier to greater openness in research may be apathy in many research communities. Many academic researchers either disagree on or are unaware of the importance (and benefits) of working openly. Since they were not trained in doing this, e.g., during graduate school or during postdoctoral training, they also may simply be unaware of how to research openly, or the resources that are available to do so. Furthermore, since most of their colleagues, collaborators, and competitors do not practice open science, no pressure comes from the research community to change. In addition, some communities do not support or actively oppose activities such as submitting preprints. These challenges seem to be particularly prevalent in engineering, especially when compared with some sub-disciplines of physics where accessibility of scholarship is markedly higher ^18^. It could be that the industry connections and strongly applied nature of engineering have hindered adoption of open practices.

This lack of pressure is related to the other major issue: lack of institutional recognition and reward for open practices. Many academic researchers will focus on what gets them credit for promotion and tenure—anything beyond that requires strong intrinsic motivation, or external motivators from the research community. At most institutions, promotion and tenure review includes some judgment (whether explicit or implicit) of where faculty publish their work, but many, “high-impact” traditional publication venues—particularly domain journals—may not support, e.g., the posting of preprints. Along with pushing publishers to support the posting of preprints, progress may be made by reminding researchers about the citation advantages of open access publishing and open dissemination of data, software, and hardware ^9, 17, 22– 24^.

## Recommendations for university leaders

As already discussed, there are real career advantages to open-access publishing and open dissemination of data, code, or other research products and therefore, for some, the incentives to conduct open research may already be in place. However, for many, citation metrics alone are not enough to ensure success in promotion and tenure, and therefore they must play to the norms of their field, department, and institution. Therefore, the institution (and the department) should look to institute policy that redefines how we measure success in academic engineering research. Some suggestions include focusing less on journal-level metrics and lending greater credibility to article-level metrics. For article-level metrics, go beyond the citation count and look for other evidence of research impact such as alternative metrics (tweets, blog posts, media coverage) and replication by others. Lastly, look for evidence of broader implications such as economic development, student development, or even lives saved. Encourage your researchers to aim for those broader impacts and value them greater than the publishing of *one more paper*. These broader impacts have real benefits in terms of institutional reputation, particularly among the general public where perception of the institution’s value can be tied to this increased societal impact.

Thinking about what institutions can do to promote open engineering research and create support structures around open dissemination, we provide the following recommendations:

- Require research products to be made openly available and then support this requirement by having a high-quality institutional repository, supporting other open repositories, and lobbying publishers to modify their copyright policies to promote the publishing of preprints and other products prior to journal submission as well as archiving of final version manuscripts.
- Convert technology commercialization offices into research impact offices. Use these offices as a mechanism for helping researchers broaden their impact through open research best practices, for funding social entrepreneurship, and for advocating these institutional activities at the state, national, and international levels.
- Empower and fund our university libraries to help with open knowledge dissemination. Others have described ways in which research outputs can be pushed public in real time with the support of the library ^47^, institutions should promote and support these efforts.
- Educate our undergraduate and graduate students on the importance of open knowledge dissemination and the practices that support it. Create and sponsor workshops that train participants in open-source software development, open research dissemination, and global development. Many institutions embrace service learning as a mechanism for greater civic engagement ^48^—broaden this approach in a thoughtful and impactful manner, being careful to ensure that students are learning the right lessons and that partnering communities are not unduly burdened ^49^. These approaches can help ensure that young engineers remain passionate about the field and hold onto the core societal mission of engineering ^50^.

Thinking specifically about the perspective of the researcher within an institution, the following list of recommendations for departments are mostly targeted at changing criteria for promotion and tenure, and performance reviews, to encourage faculty to practice more open science:

- Consider accessibility/openness of research products along with quantity and “quality” in promotion and tenure review. Mandate self-archiving of publications (i.e., green open access).
- Recognize research products such as software and data, and their associated impacts (e.g., citations), as equal to traditional publications in scholarly impact.
- Reduce the importance of publishing in traditional venues for promotion and tenure, recognizing these may be barriers to openness.
- Provide educational opportunities that train faculty and other researchers in open science skills, and those necessary to work with software and data.

Research communities that impede openness cannot be forced to change from the outside. Instead, by making changes to institutional reward systems, researchers will be encouraged to improve their open practices, and thus evolve communities from the inside.

## Conclusions

In this paper we have reviewed the existing state of knowledge on the benefits and challenges of practicing openness in engineering research. We have further briefly outlined our thoughts on how open research practices in the sciences, engineering, and other fields can and should be employed by public universities to position themselves as centers for the creation and broad dissemination of knowledge as a public resource. Resistance to this proposal is prevalent through reluctance to change and, in some cases, apathy on the part of researchers. Additionally, many researchers operate in an environment that devalues an educated populace and with systemic practices and policies that exclusively reward the monetization of any form of intellectual property. Change likely needs to be driven with grassroots initiatives that demonstrate the possible benefits and make it clear that tax dollars could fund these efforts if distributed properly and with accountability. However, many of the recommendations provided here would require little or no additional funding as the mechanisms that would enable them, such as recognition of diverse research products in hiring and tenure and promotion criteria, already exist as part of normal academic routines.

## Footnotes

^a^“If I have seen further, it is by standing on the shoulders of giants.” Isaac Newton (1676), although similar statements can be found as far back as the 12th century.

^b^For example, the Open Definition: https://opendefinition.org/.

^c^Of course, papers that are read may not be cited, and papers that are cited may not actually be read.

^d^ https://engrxiv.org

^e^ https://figshare.com

^f^ https://zenodo.org

^g^ https://creativecommons.org/licenses/by/4.0/legalcode

^g^A listing of research data repositories can be found at https://www.re3data.org/

^i^ http://joss.theoj.org/

^j^ https://www.tjoe.org/

^k^NIH Public Access Policy https://publicaccess.nih.gov/index.htm

^l^NSF Public Access Plan https://www.nsf.gov/pubs/2015/nsf15052/nsf15052.pdf

^m^Bill & Melinda Gates Foundation Open Access Policy http://www.gatesfoundation.org/How-We-Work/General-Information/Open-Access-Policy

^n^Wellcome Trust Open Access Policy https://wellcome.ac.uk/funding/managing-grant/open-access-policy

^o^ROARMAP http://roarmap.eprints.org/

^p^SPARC Author Addendum https://sparcopen.org/our-work/author-rights/#addendum

^q^Zenodo currently accepts datasets up to 50 GB, but stores data in the CERN Data Center, along with 100 PB of physics data from the Large Hadron Collider ( https://help.zenodo.org/).
