# Supplementary material for: The case for openness in engineering research
Source: F1000Res. 2018 Oct 11;7:501. Originally published 2018 Apr 26. [Version 2] doi: 10.12688/f1000research.14593.2 (PMC6733375; doi:10.12688/f1000research.14593.2)
Supplement: Peer review history

# Peer review: Reviewer response for version 2

**DOI:** 10.5256/f1000research.18090.r53346

**Published:** 2019-09-10

**Authors:** Chris H. J. Hartgerink

**Affiliations:** 1 Liberate Science, Berlin, Germany

I read "The case for openness in engineering research" and found it to provide a good overview of the state of openness in general.

 My main remark for the authors is that I felt like I was familiar with the contents of the opinion piece, despite having zero knowledge about engineering research. Maybe the case for openness in engineering research is simply the case for openness, give a few field-specific details? I'd expected more differentiation, given the statement that it would be focused on engineering. All in all, I found little to nothing to actively disagree with in respect to factual claims.

 One particular aspect that might be specific to engineering research are patents. Public disclosure can preclude an invention from patenting, if it takes longer than 12 months for the patent to be filed after initial public disclosure of the invention. However, patents are relatively rarely discussed in the open space compared to licensing, so I don't know whether this would fit in or not. It might also be relevant to refer the "Patent trolling" scenario to the authors for this.

 https://www.eff.org/reclaim-invention/pledge

 Some minor aspects:

- In the "Accessibility:" paragraph, licensing isn't mentioned. For something to remain accessible, permissive licensing is necessary (otherwise it may disappear at any point).
- "Reproducibility:" helps understand, not enable, reproducibility in my perspective but I'm nit-picking (this is a good sign because there's hardly anything else to comment on).
- "Establish priority:" is a good section. I usually refer to this as the Scooping Paradox, which helps people remember. Never wrote it down, so if you like it, it's yours.
- Regretfully, PeerJ preprints will stop accepting new preprints September 30th, which may (or may not) affect the authors' willingness to include it as a recommendation.
  https://twitter.com/jasonHoyt/status/1168809204062269442

I confirm that I have read this submission and believe that I have an appropriate level of expertise to confirm that it is of an acceptable scientific standard.

# Peer review: Reviewer response for version 2

**DOI:** 10.5256/f1000research.18090.r53347

**Published:** 2019-09-06

**Authors:** Patrick Vandewalle

**Affiliations:** 1 EAVISE, Department of Electrical Engineering (ESAT), KU Leuven, Leuven, Belgium

The authors make a clear case for more openness in engineering research. They give a well-balanced set of arguments for researchers to perform open research, and argue that it increases the societal impact of the research. They also discuss potential obstacles that researchers may encounter when making their work more open. Finally, they also give a set of recommendations for university leaders to improve and support openness. 

 I have some minor questions/comments, which I hope can help in further strengthening the points made in the article. 

1. The funding paragraph in the introduction is currently very much focused on US public universities, which of course does not cover the entire readership. 
2. The arguments provided in this article mostly hold for publicly funded academic research in engineering. A lot of engineering research is also performed in industry, or at universities (partially) funded by industry. In such projects, contracts often do not allow full openness of publication, code and data. It could be a useful addition to mention this (more) clearly in the paper. 
3. Regarding my work on citation analysis of image processing papers with and without code, I recently presented an update of this analysis at the WIC Symposium (see Vandewalle, 2019 ^1^). Just providing this information for your reference. 
4. I do not fully agree with the argument of establishing priority by releasing preprints to avoid being "scooped". This is true, but when researchers fear to be "scooped", I believe they generally fear that others will be faster in their further exploration of a data set or a piece of code and hence do not allow the original authors to take full advantage of their initial efforts. Meanwhile, I believe the risk of being "scooped" is generally overestimated and does (in most cases) not provide a real threat. 
5. In the section "It's nice", the authors argue that it prevents unnecessary repetition of work. I fully agree with this argument, and see this improved efficiency as one of the strong benefits of more openness. Meanwhile, there are also some advantages to such an independent repetition of an experiment, as it reduces the risk of repeating or overlooking the same bugs. It could be useful to add a comment in that sense to the article to keep arguments well-balanced. 
6. Finally, in the introduction to "Open science and the scholar's research agenda", the authors mention that "sometimes it is enough to simply be more open than the current norms in their field." While this makes sense for researchers wanting to get some benefits of being open by doing somewhat better than their peers, I believe we should set sufficiently high standards for open research, and at least aim for full openness where possible.

As already mentioned above, these comments were mostly my views about some aspects of the present opinion article. In general, I think it is already a well-written article and I would like to thank the authors for their efforts in putting this opinion article together to further promote open science.

I confirm that I have read this submission and believe that I have an appropriate level of expertise to confirm that it is of an acceptable scientific standard.

## References

[1] : Code availability for image processing papers: a status update. *WIC IEEE SP Symposium on Information Theory and signal Processing in the Benelux*.2019; Reference source

# Peer review: Reviewer response for version 2

**DOI:** 10.5256/f1000research.18090.r39354

**Published:** 2018-10-12

**Authors:** Nathan L. Vanderford

**Affiliations:** 1 Markey Cancer Center, Department of Toxicology and Cancer Biology, College of Medicine, University of Kentucky, Lexington, KY, USA

The authors have revised the article to address most of my prior comments/concerns and that of Dr. Pearce, the other official reviewer. In particular, the addition of references, addition of text (e.g., a new “value and credit” subsection), and wording revisions have strengthened the work. The article is an important contribution to the literature on open science. The article should promote additional dialogue on this important topic.

I confirm that I have read this submission and believe that I have an appropriate level of expertise to confirm that it is of an acceptable scientific standard.

# Peer review: Reviewer response for version 1

**DOI:** 10.5256/f1000research.15880.r33671

**Published:** 2018-05-04

**Authors:** Joshua M. Pearce

**Affiliations:** 1 Department of Electrical and Computer Engineering, Michigan Technological University, Houghton, MI, UK

This is an important paper - and sadly still needs to be written in 2018 - as open science should clearly be the default for the maximum rate of progress in any field. It is clear that we are headed this way but the rate of change could certainly be faster. In addition to the comments from the other reviewers I offer the following constructive points:

1. Although this is an opinion piece I would encourage the authors to avoid all unsubstantiated claims. Ideally every fact not derived from the authors' own work should have a citation.
2. Although I agree with the first line - the article would be stronger if the footnote offered either a substantial list of referenced arguments for it -- or wait until the end to make the claim.
3. Instead of simply saying open source software - it would be better to use FOSS (and explain the difference).
4. To be correct in the definition of open science - the use of free and open source hardware should be included in addition to FOSS.
5. I am not sure that open science is benefited by "the half-baked ideas, the napkin sketches, the first drafts, and the failures." For example, engineers working on water purification technology should not publish their tech until they are sure it works unless it has warning notices all over it.
6. It would be interesting to speculate on why engineers lag so far behind say physicists in making their work open access. Is it because the various engineering societies have more restrictive publishing agreements than the major publishers?
7. There are some examples of method to quantify the impact of research on society. There is a rich literature showing high ROIs for industry funded research for the business world. In addition using the concept of downloaded substitution value one can calculate the value to society for open source scientific hardware designs as well as software.
8. Increasing societal impact of research + you make a good point about the scientists in the Global South - but you should consider going one step further and encouraging engineers working on technologies that can solve the problems of the world's poorest people to make sure they are released as open source appropriate technologies.
9. Impact - you can make a stronger case. Patenting slows innovation (and there are a ton of studies showing this) and increases costs for consumers. One of the most clear recent examples is the staggering decrease in costs and increases in performance of 3D printers following the open source release of the RepRap project.
10. Engineering journals + most of them do offer some sort of open access policy - either to post preprints or pay for open access.
11. Open workflow should encourage the use of FOSS and FOSH whenever possible.
12. pg 5 seems overly pessimistic and also does not cite proof. I have found that most researchers are sympathetic to open science and that many forward-thinking institutions are pushing open access pretty hard.
13. Even just going to article level metrics - where OA has an advantage for citations should be useful and already has a built in incentive.
14. Something should be said about open source business models. Patents are not the only way to go -- i.e. RedHat is a multi-billion per year open source company.
15. Finally, many of your suggestions are good but could be strengthened if you hone in on university leaders self interest for encouraging them to actually implement them.

I confirm that I have read this submission and believe that I have an appropriate level of expertise to confirm that it is of an acceptable scientific standard, however I have significant reservations, as outlined above.

# Response

**Published:** 2018-10-08

**Authors:** Devin Berg

**Affiliations:** University of Wisconsin, Stout, USA

Dr. Pearce,

Thank you for your thorough review. We agree that open science practices should indeed be the default for engineering research and progress. We'd like to address your individual points below.

*1) Although this is an opinion piece I would encourage the authors to avoid all unsubstantiated claims. Ideally every fact not derived from the authors' own work should have a citation.*

We have gone through the paper again and attempted to address any issues with uncited statements. Several new citations and references have been added.

*2) Although I agree with the first line - the article would be stronger if the footnote offered either a substantial list of referenced arguments for it -- or wait until the end to make the claim.*

The first line is stated as an opinion, which we intend to use as the basis for the rest of the paper. This statement is fleshed out more fully with references to other related works in the following paragraphs.

*3) Instead of simply saying open source software - it would be better to use FOSS (and explain the difference).*

We have added language to make this distinction.

*4) To be correct in the definition of open science - the use of free and open source hardware should be included in addition to FOSS.*

Agreed. We have added hardware to this definition.

*5) I am not sure that open science is benefited by "the half-baked ideas, the napkin sketches, the first drafts, and the failures." For example, engineers working on water purification technology should not publish their tech until they are sure it works unless it has warning notices all over it.*

We agree with the first sentence here and have modified the language to word this differently. For the second sentence, we agree that there needs to be more education on what early work is and its use cases. However, we don't feel that this is limited to any one sub-discipline such as "water purification technology" and is actually an issue that applies to virtually all of engineering.

*6) It would be interesting to speculate on why engineers lag so far behind say physicists in making their work open access. Is it because the various engineering societies have more restrictive publishing agreements than the major publishers?*

This is a difficult question to answer. Perhaps there are cultural limitations due to the applied or industry connected nature of engineering. We've added some discussion to the "Challenges" section to try to address this better.

*7) There are some examples of method to quantify the impact of research on society. There is a rich literature showing high ROIs for industry funded research for the business world. In addition using the concept of downloaded substitution value one can calculate the value to society for open source scientific hardware designs as well as software.*

We have added some discussion and relevant citations to the societal impact section of the paper.

*8) Increasing societal impact of research + you make a good point about the scientists in the Global South - but you should consider going one step further and encouraging engineers working on technologies that can solve the problems of the world's poorest people to make sure they are released as open source appropriate technologies.*

Agree and we have added some additional discussion to this section of the paper.

*9) Impact - you can make a stronger case. Patenting slows innovation (and there are a ton of studies showing this) and increases costs for consumers. One of the most clear recent examples is the staggering decrease in costs and increases in performance of 3D printers following the open source release of the RepRap project.*

Have added some discussion and references to support this.

*10) Engineering journals + most of them do offer some sort of open access policy - either to post preprints or pay for open access.*

We have to generally disagree with this point. Several of the larger engineering societies are lagging in explicit support of preprinting. Additionally, we don't view hybrid OA journals as a solution to this problem as they are largely shifting the fees and associated publisher profits from one hand to the other. We view hybrid OA journals as a temporary solution on the path towards full OA journals with reasonable APCs operated by societies or other non-profit academic institutions.

*11) Open workflow should encourage the use of FOSS and FOSH whenever possible.*

Have edited the language to match.

*12) pg 5 seems overly pessimistic and also does not cite proof. I have found that most researchers are sympathetic to open science and that many forward-thinking institutions are pushing open access pretty hard.*

We have edited the language here. Also, it seems that these forward-thinking institutions are pushing OA publishing but not open science.

*13) Even just going to article level metrics - where OA has an advantage for citations should be useful and already has a built in incentive*.

True, we have reiterated this point.

*14) Something should be said about open source business models. Patents are not the only way to go, RedHat is a multi-billion per year open source company.*

This is true, though we've tried to focus this paper more on research than industry/innovation.

*15) Finally, many of your suggestions are good but could be strengthened if you hone in on university leaders self interest for encouraging them to actually implement them.*

Have edited language to reiterate the benefits of self-interest.

# Peer review: Reviewer response for version 1

**DOI:** 10.5256/f1000research.15880.r33494

**Published:** 2018-04-30

**Authors:** Nathan L. Vanderford

**Affiliations:** 1 Markey Cancer Center, Department of Toxicology and Cancer Biology, College of Medicine, University of Kentucky, Lexington, KY, USA

This is a well-written opinion article that presents a well-articulated argument for the science community to increase the practice of “openness” in research. After clearly defining  “open” research (caveat below), key components of the article include a discussion on the importance of open research to science, some recommendations on how researchers can conduct their research “openly” and how universities can support such work, and there is a discussion about challenges to open science. The article is a valuable contribution to this growing movement.

 Although the article is well written and the argument is clearly articulated with appropriate references to the literature, I believe that there are a few points that the authors should consider and respond to prior to this work being approved for indexing.

1. The authors reference the work as a “review” but in the parlance of F1000Research, it is an opinion article and it is written as such since it is an argument for conducting more open research. Thus, this reviewer suggests not referring to the work as a “review.”
2. The article title and a few areas within the text are written specifically toward engineering research, but this topic transcends disciplines and much of the content can be applied broadly. As such, the authors should consider whether the title and respective text should be less restricted to engineering. Perhaps the authors could present the topic as one that is more broadly applicable but then more clearly state that they are using engineering as a case study/example to illustrate their points.
3. The authors begin the main text with a clear definition of open science, but it is unclear if this is the authors’ definition or if it is taken from the literature. This should be clarified.
4. The authors should consider adding a bit more discussion on the challenges associated with open research. For example, more could be said about how the culture of academia doesn’t particularly value open research currently. Today’s academic culture is still too focused on where a journal is ranked based on impact factor and this plays into aspects of faculty life such as faculty hiring, promotion/tenure, faculty performance evaluations, post-tenure review, etc.  While the authors touch on some of these points, a greater discussion and recommendation for a culture change could be a valuable, thought-provoking addition to the article. More could also be said regarding some of the minutia of how open research needs to be implemented especially related to the infrastructure needed to support it and the financial costs associated with the infrastructure. Who should pay for hosting, pay publications costs, etc.?

In summary, this is a well-articulated article on an important, timely topic. The few points above slightly dampen this reviewer’s enthusiasm at this time related to giving full approval. As such, I look forward to reviewing a revised version of the article.

I confirm that I have read this submission and believe that I have an appropriate level of expertise to confirm that it is of an acceptable scientific standard, however I have significant reservations, as outlined above.

# Response

**Published:** 2018-10-05

**Authors:** Devin Berg

**Affiliations:** University of Wisconsin, Stout, USA

Dr. Vanderford,

Thank you for your thoughtful review of our paper and for your specific suggestions for improvement. We've attempted to address your comments in this revision as outlined below.

*1. The authors reference the work as a “review” but in the parlance of F1000Research, it is an opinion article and it is written as such since it is an argument for conducting more open research. Thus, this reviewer suggests not referring to the work as a “review.”*

We've revised the language to make it more clear that this is not a review article and more of an opinion article.

*2) The article title and a few areas within the text are written specifically toward engineering research, but this topic transcends disciplines and much of the content can be applied broadly. As such, the authors should consider whether the title and respective text should be less restricted to engineering. Perhaps the authors could present the topic as one that is more broadly applicable but then more clearly state that they are using engineering as a case study/example to illustrate their points.*

We agree that many of the issues that we've addressed are not unique to engineering and do indeed apply more broadly. However, our goal with this work was to write a targeted paper for the engineering community that specifically addresses the issues from that frame. There are other works in the literature that take a more general approach and we've tried to reference them appropriately to bring them to the reader's attention.

*3) The authors begin the main text with a clear definition of open science, but it is unclear if this is the authors’ definition or if it is taken from the literature. This should be clarified.*

The definition of open science included in our paper is a synthesis of other available definitions found elsewhere. We've edited to make this more clear and inserted some references to support this.

*4) The authors should consider adding a bit more discussion on the challenges associated with open research. For example, more could be said about how the culture of academia doesn’t particularly value open research currently. Today’s academic culture is still too focused on where a journal is ranked based on impact factor and this plays into aspects of faculty life such as faculty hiring, promotion/tenure, faculty performance evaluations, post-tenure review, etc. While the authors touch on some of these points, a greater discussion and recommendation for a culture change could be a valuable, thought-provoking addition to the article. More could also be said regarding some of the minutia of how open research needs to be implemented especially related to the infrastructure needed to support it and the financial costs associated with the infrastructure. Who should pay for hosting, pay publications costs, etc.?*

We have addressed issues around a lack of valuation for open science practices and the need for change in promotion/tenure evaluation criteria. We've tried to emphasize this more in the paper. We would also point out that many of our recommendations do not require financial support to be feasible and indeed would be possible under existing funding models.
